# Circulatory CCL2 distinguishes Duchenne muscular dystrophy dogs

**DOI:** 10.1242/dmm.052137

**Published:** 2025-03-14

**Authors:** Dennis O. Pérez-López, Matthew J. Burke, Chady H. Hakim, James A. Teixeira, Jin Han, Yongping Yue, Zewei Ren, Jianguo Sun, Shi-jie Chen, Roland W. Herzog, Gang Yao, Dongsheng Duan

**Affiliations:** ^1^ Department of Molecular Microbiology and Immunology, School of Medicine, University of Missouri, Columbia, MO 65212, USA; ^2^ Department of Statistics, University of Missouri, Columbia, MO 65212, USA; ^3^ Department of Physics and Astronomy, University of Missouri, Columbia, MO 65211, USA; ^4^ Department of Biochemistry, University of Missouri, Columbia, MO 65211, USA; ^5^ MU Institute for Data Science and Informatics, University of Missouri, Columbia, MO 65211, USA; ^6^ Herman B Wells Center for Pediatric Research, Indiana University, Indianapolis, IN 46202, USA; ^7^ Department of Chemical and Biomedical Engineering, College of Engineering, University of Missouri, Columbia, MO 65212, USA; ^8^ Department of Neurology, School of Medicine, University of Missouri, Columbia, MO 65212, USA; ^9^ Department of Biomedical Sciences, College of Veterinary Medicine, University of Missouri, Columbia, MO 65212, USA

**Keywords:** Duchenne muscular dystrophy, Canine model, CCL2, Cytokines/chemokines, DMD, Muscle inflammation

## Abstract

To establish a minimally invasive approach to studying body-wide muscle inflammation in the canine Duchenne muscular dystrophy (DMD) model, we evaluated 13 cytokines/chemokines in frozen sera from 90 affected (239 sera) and 73 normal (189 sera) dogs (0.00 to 45.2 months of age). Linear mixed-effects model analysis suggested that ten cytokines/chemokines were significantly elevated in affected dogs, including interleukin (IL)-2, IL-6, IL-7, IL-8, IL-10, IL-15, IL-18, C-C motif chemokine ligand 2 (CCL2), C-X-C motif chemokine ligand 1 (CXCL1) and granulocyte-macrophage colony-stimulating factor (GM-CSF). Further, cytokine/chemokine elevation coincided with the onset of muscle disease. Importantly, only CCL2 showed consistent changes at all ages, with the most pronounced increase occurring between 3 and 9 months. To study the effects of sample storage and type, we compared fresh versus frozen, and serum versus plasma, samples from the same dog. Similar readings were often obtained in fresh and frozen sera. Although plasma readings were significantly lower for many cytokines/chemokines, this did not compromise the robustness of CCL2 as a biomarker. Our study establishes a baseline for using circulatory cytokines/chemokines as biomarkers in canine DMD studies.

## Teaser

**Summary:** We identified CCL2 as a reliable serum biomarker in the canine model of Duchenne muscular dystrophy (DMD), establishing a baseline for using circulatory cytokines/chemokines as biomarkers in canine DMD studies.

## INTRODUCTION

Duchenne muscular dystrophy (DMD) is a severe progressive X-linked myopathy that affects 1 in 5000 to 1 in 6000 live male births (Duan et al., 2021). DMD is caused by null variants in the *DMD* gene, which encodes dystrophin, a critical structural protein in muscle (Kunkel, 2005). Affected boys show signs of delayed motor development between 2 and 5 years of age (Birnkrant et al., 2018). Patients lose ambulation in their early teens and die from respiratory and/or cardiac complications before the age of 40 years. Currently, there is no cure for DMD.

Dystrophin-deficient animals were identified soon after the cloning of the *DMD* gene (Cooper et al., 1988; Sicinski et al., 1989). Hundreds of DMD animal models have been reported in different species, including mice, rats, rabbits, pigs, dogs and non-human primates (McGreevy et al., 2015; Wasala et al., 2020). Preclinical studies in these models have established the foundation for human trials to test pharmacological and genetic therapies. Most preclinical studies are performed in murine DMD models. Unfortunately, many promising results in mice have failed to deliver clinical benefits in human patients (Birmingham, 1997; Mendell et al., 1995; Phillips and Quinlivan, 2008; Satoyoshi, 1992; Wagner et al., 2008). The lack of validation in a large-animal DMD model contributes to the translation failure (Duan, 2011).

The canine model is the first and most used large-animal model in DMD research (Cooper et al., 1988). Affected dogs recapitulate many aspects of human disease (Duan, 2015; Kornegay, 2017). Although the canine model has been used to test drug therapy (Barraza-Flores et al., 2019; Kornegay et al., 2014; Liu et al., 2004), cell therapy (Sampaolesi et al., 2006), exon-skipping therapy (Echigoya et al., 2017; Yokota et al., 2009), microdystrophin gene therapy (Birch et al., 2023; Le Guiner et al., 2017; Shin et al., 2013; Yue et al., 2015), dystrophin-independent gene therapy (Kodippili et al., 2024; Song et al., 2019) and CRISPR editing therapy (Amoasii et al., 2018; Hakim et al., 2021), our understanding of canine DMD models remains limited.

A major function of dystrophin is to stabilize the sarcolemma during muscle contraction. The absence of dystrophin results in contraction-induced muscle cell degeneration and necrosis and, consequently, profound inflammatory responses (Grounds et al., 2020; Reid and Alexander, 2021; Rosenberg et al., 2015; Tripodi et al., 2021; Tulangekar and Sztal, 2021). Many studies have examined muscle inflammation in the canine DMD model by histological and immunohistological staining of tissues collected at biopsy and necropsy (Birch et al., 2023; Kodippili et al., 2024; Le Guiner et al., 2017; Shin et al., 2013; Yue et al., 2015). However, biopsy is invasive, and necropsy requires the termination of the animal. Further, these assays can only reveal inflammation in a small area of a limited number of muscles. To develop a minimally invasive approach for studying body-wide inflammatory changes in the canine DMD model, we examined blood cytokines/chemokines using a canine-specific Luminex assay. A total of 13 cytokines/chemokines were evaluated, including interleukin (IL)-2, IL-6, IL-7, IL-8 (also known as CXCL8), IL-10, IL-15, IL-18, CC-motif chemokine ligand 2 [CCL2; also known as monocyte chemoattractant protein 1 (MCP-1)], CXC-motif ligand 1 [CXCL1; also known as keratinocyte chemotactic-like (KC-like)], CXCL10 [also known as IFN-γ-induced protein 10 (IP-10)], granulocyte-macrophage colony-stimulating factor [GM-CSF; also known as colony-stimulating factor 2 (CSF2)], interferon-gamma (IFN-γ) and tumor necrosis factor-alpha [TNF-α; also known as tumor necrosis factor (TNF)]. We characterized their circulatory concentrations in frozen serum collected from a large cohort of normal and affected dogs at different ages. We further examined whether sample storage (fresh versus frozen) and sample type (serum versus plasma) influenced the results. Our studies lay the foundation for using circulatory cytokines/chemokines as biomarkers in studying DMD pathogenesis and evaluating experimental therapeutics.

## RESULTS

### Overview of serum samples used in the analysis

To determine disease-, age- and sex-associated changes in circulatory cytokines/chemokines, we studied 428 frozen serum samples collected from 163 dogs. Of these, 189 samples were from 73 normal dogs (0.00 to 41.03 months of age), and 239 samples were from 90 affected dogs (0.03 to 45.2 months of age). Serum samples were collected once from 27 normal dogs. In the remaining normal dogs, multiple serum samples were collected from the same dog at different time points (Fig. S1A). Serum samples were collected once from 47 affected dogs. For the remaining affected dogs, multiple serum samples were collected from the same dog at different time points (Fig. S1A).

### Overview of the cytokine/chemokine measurement results

We evaluated the distribution of all measurement results (Fig. S1B). In general, lower values were more frequently observed in serum collected from normal dogs, whereas higher values were more frequently found in serum obtained from affected dogs (Fig. S1B). The measurements below the detection limit were assigned as 0 pg/ml (undetectable). The zero value was more frequently seen in normal dogs than in affected dogs (Fig. S1B, Table S1). The logistic linear mixed-effects model confirmed that such differences reached statistical significance for IL-2, IL-6, IL-7, IL-10, IL-15, IL-18 and GM-CSF (Table S1). Notably, a high percentage of measurements were below the detection limits for IL-2 (63% normal and 34% affected), IL-15 (51% normal and 30% affected) and TNF-α (91% normal and 85% affected) (Table S1). The undetectable measurements were not seen or rarely seen for IL-8 (0.5% normal and 0.0% affected), CCL2 (0.0% normal and 0.0% affected) and CXCL1 (0.0% normal and 0.8% affected) (Table S1).

**Fig. 1.**
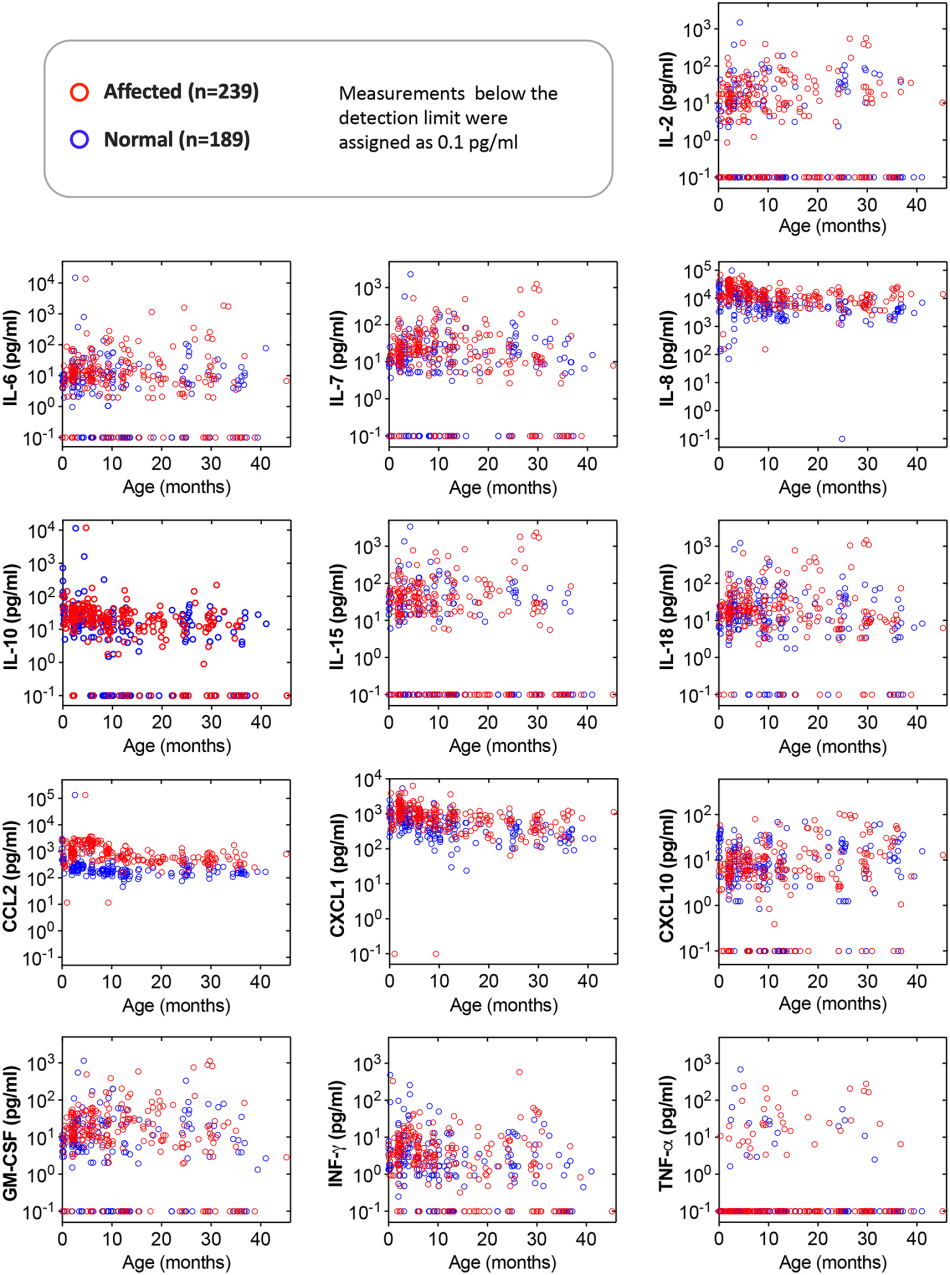
**Overview of cytokine/chemokine levels in normal and affected dogs.** Scatter plots show log-transformed cytokine/chemokine values from all study samples. Each circle represents the data from one sample. The 0 pg/ml value (below the detection limit) was replaced with 0.1 pg/ml in log transformation.

For measurements above the detection limit, we noted that the data range was quite large (over several logs). To visualize cytokine/chemokine measurement results of all 428 serum samples, we plotted the data in the log_10_ scale, and we also used 0.1 pg/ml to replace measurements below the detection limit (0 pg/ml) in graphs so that they could be visualized in the log-scale plot (Fig. 1). The value 0.1 pg/ml is lower than the lowest measured value (0.25 pg/ml) in the entire dataset (Table S1). The concentration differences between normal and affected dogs were apparent for three cytokines/chemokines: IL-8, CCL2 and CXCL1. These three cytokines/chemokines appeared elevated in affected dogs (Fig. 1). No apparent trends were visible for other analytes (Fig. 1).

### Multiple cytokines/chemokines are significantly elevated in serum collected from affected dogs

**Fig. 2.**
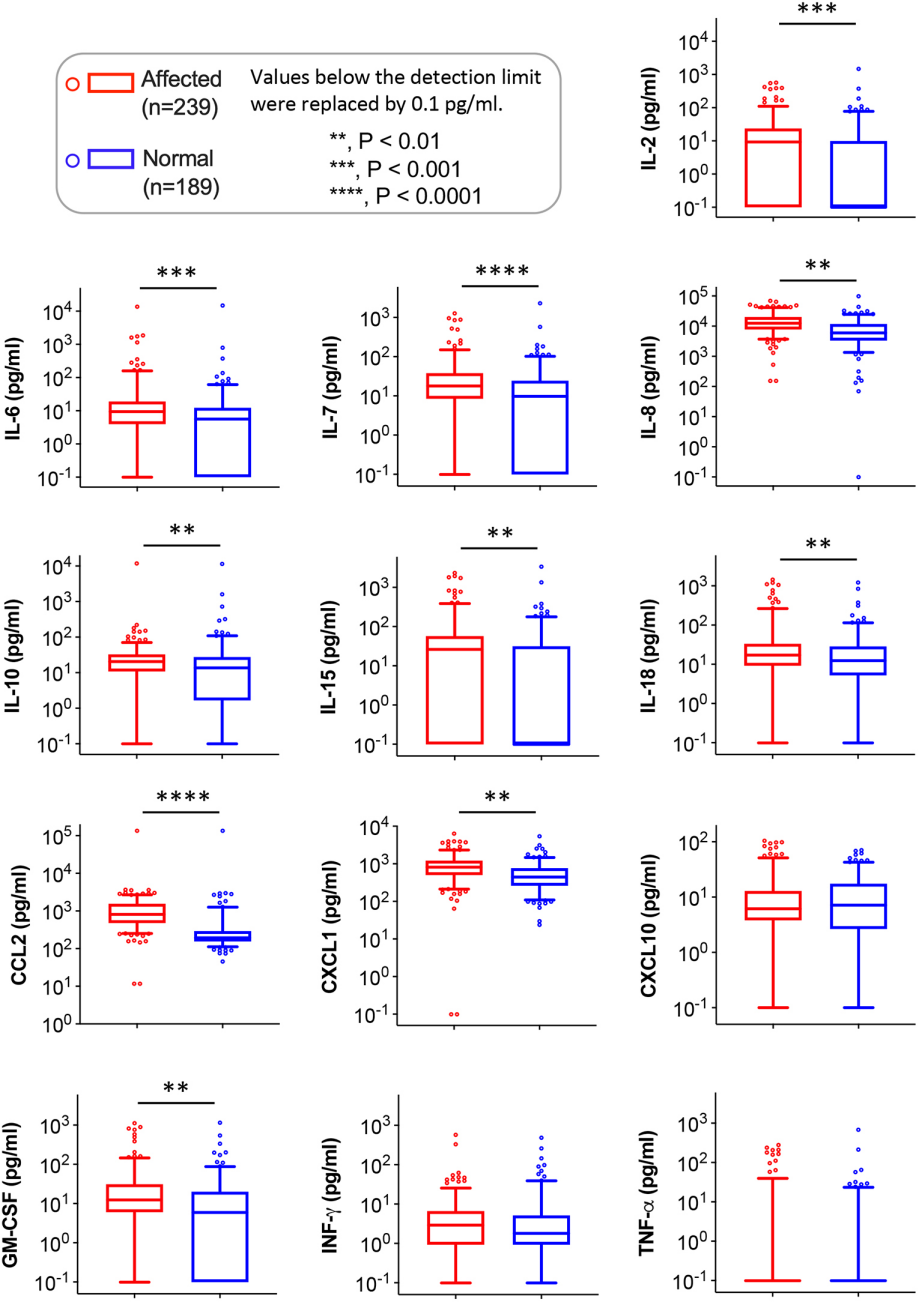
**Comparison of cytokine/chemokine levels between normal and affected dogs.** Box plots are shown in logarithmic scale owing to the large data range, and 0.1 was added to visualize zero values. The effects of genotype (normal versus affected) were analyzed using the linear mixed-effects model. The box spans from the 25th (Q1) to 75th (Q3) percentiles, representing the middle 50% of the data. The line inside the box marks the median (50th percentile). The upper whisker marks the 95th percentile. The lower whisker marks the fifth percentile. Values beyond the fifth and 95th percentiles are considered outliers and depicted as individual circles. **P<0.01; ***P<0.001; ****P<0.0001.

We analyzed the genotype (normal versus affected) effects on log-transformed cytokines/chemokines concentrations using the linear mixed-effects model. Ten analytes were significantly elevated in affected dogs, including IL-2, IL-6, IL-7, IL-8, IL-10, IL-15, IL-18, CCL2, CXCL1 and GM-CSF (Fig. 2). Table S2 shows the descriptive statistic values of the entire dataset, including minimum, maximum, mean, s.e.m., median, 25th percentile quartile (Q1), 75th percentile quartile (Q3) and confidence interval (CI).

### Multiple cytokines/chemokines are significantly elevated in serum collected from affected dogs at different ages

**Fig. 3.**
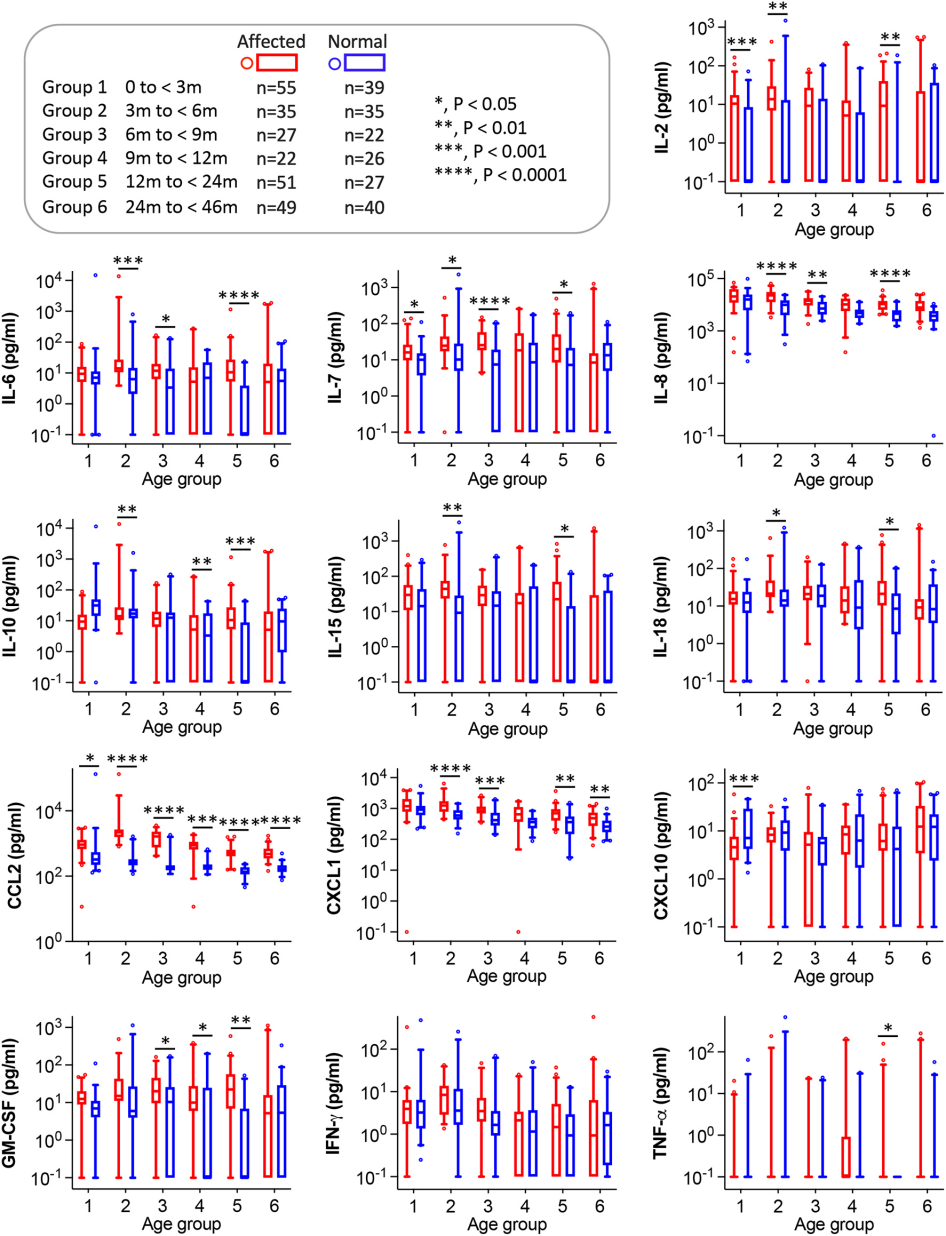
**Age-based comparison of cytokine/chemokine levels between normal and affected dogs.** Serum concentrations of cytokines/chemokines are compiled into one of the six age groups based on the age at the time of serum collection. Box plots are shown in logarithmic scale owing to the large data range, and 0.1 was added to visualize zero values. The effects of genotype (normal versus affected) were analyzed using the linear mixed-effects model in each age group. The box spans from the 25th (Q1) to 75th (Q3) percentiles, representing the middle 50% of the data. The line inside the box marks the median (50th percentile). The upper whisker marks the 95th percentile. The lower whisker marks the fifth percentile. Values beyond the fifth and 95th percentiles are considered outliers and depicted as individual circles. *P<0.05; **P<0.01; ***P<0.001; ****P<0.0001. m, months.

To further compare normal and affected dogs, we grouped dogs into six age groups, including group 1 (<3 months), group 2 (3 to <6 months), group 3 (6 to <9 months), group 4 (9 to <12 months), group 5 (12 to <24 months) and group 6 (24 to <46 months). We analyzed the effects of genotype on cytokine/chemokine concentrations in each age group by applying the linear mixed-effects model (Fig. 3). We also calculated reference values for each age group (Table 1).

**Table 1.** Reference values for all 13 cytokines/chemokines in each age group (pg/ml)

		Cytokine	*n*	Minimum	Maximum	Mean	s.e.m.	Median	Q1	Q3	95% CI of mean	
											Lower	Upper
Age group 1 (0 to <3 months)	Normal	IL-2	33	0.0	72.3	6.6	2.4	0.0	0.0	8.8	3.0	11.5
		IL-6	33	0.0	14,781.4	458.0	447.6	7.1	5.0	10.8	7.7	1355.8
		IL-7	33	0.0	110.5	14.3	3.4	10.1	6.2	15.2	9.1	21.9
		IL-8	33	112.5	97,723.7	19,569.4	2983.4	16,733.9	10,509.5	24,723.3	14,518.2	26,232.4
		IL-10	33	5.0	11,564.8	417.1	349.1	31.4	17.3	58.0	39.2	1140.0
		IL-15	33	0.0	294.1	40.0	11.1	20.6	8.8	43.6	21.6	65.2
		IL-18	33	0.0	178.0	20.6	5.3	13.5	6.7	24.5	12.9	32.9
		CCL2	33	129.0	134222.4	4776.7	4048.3	310.9	232.3	584.3	501.7	12966.9
		CXCL1	33	221.6	5419.1	1177.9	176.3	944.7	603.4	1297.5	886.3	1546.3
		CXCL10	33	2.2	38.6	14.2	2.0	11.3	4.3	24.8	10.7	18.1
		GM-CSF	33	0.0	110.1	11.7	3.3	7.8	4.0	11.2	7.1	19.0
		IFN-γ	33	0.2	291.2	13.9	8.8	2.4	1.4	6.4	3.4	32.5
		TNF-α	33	0.0	64.6	2.9	2.1	0.0	0.0	0.0	0.0	7.7
	Affected	IL-2	42	0.0	166.4	18.3	4.6	11.2	3.5	17.5	10.6	28.4
		IL-6	42	0.0	88.2	13.5	2.8	9.1	5.1	14.1	8.7	19.5
		IL-7	42	0.0	125.7	23.1	4.0	15.9	9.8	24.7	16.1	32.2
		IL-8	42	529.6	69,933.6	22,386.3	2144.0	20,202.6	12,421.4	29,920.4	18,622.1	26,766.1
		IL-10	42	0.0	142.8	32.4	4.2	25.3	18.6	36.3	25.1	41.7
		IL-15	42	0.0	401.8	50.1	10.9	30.0	14.4	55.7	31.9	74.1
		IL-18	42	0.0	180.5	23.9	4.7	15.5	10.5	25.1	16.2	34.1
		CCL2	42	218.7	2841.9	1050.9	98.0	893.4	643.2	1246.2	866.8	1244.2
		CXCL1	42	368.6	3905.9	1368.8	128.2	1126.2	797.7	1704.2	1141.3	1628.6
		CXCL10	42	0.0	58.5	6.8	1.4	4.4	3.1	7.1	4.6	10.0
		GM-CSF	42	0.0	50.4	17.0	2.0	11.9	7.8	21.3	13.4	21.1
		IFN-γ	42	0.0	333.2	11.9	7.8	3.5	1.7	6.1	3.5	28.3
		TNF-α	42	0.0	14.7	0.6	0.4	0.0	0.0	0.0	0.0	1.6

		Cytokine	*n*	Minimum	Maximum	Mean	s.e.m.	Median	Q1	Q3	95% CI of mean	
											Lower	Upper
Age group 2 (3 to <6 months)	Normal	IL-2	25	** **0.0	** **377.6	** **25.0	** **15.1	** **5.6	** **0.0	** **12.7	** **5.5	** **57.3
		IL-6	25	0.0	376.9	23.2	14.9	6.3	2.5	12.1	5.9	54.6
		IL-7	25	0.0	578.6	40.2	22.8	11.9	6.2	23.4	12.3	88.5
		IL-8	25	313.1	24,259.2	9586.1	1349.2	9869.3	3539.9	14,658.2	6988.3	12,226.9
		IL-10	25	0.0	124.0	23.2	4.9	17.2	10.3	26.4	15.5	34.0
		IL-15	25	0.0	1350.8	84.9	54.4	14.3	0.0	29.1	14.6	201.7
		IL-18	25	0.0	839.4	54.6	33.0	15.5	9.6	26.0	15.2	124.7
		CCL2	25	152.2	1650.3	345.5	61.5	261.1	220.8	328.8	252.6	476.8
		CXCL1	25	155.7	1454.2	705.5	69.8	628.6	469.7	821.7	581.4	840.0
		CXCL10	25	0.0	45.6	12.3	2.0	9.7	5.7	18.1	8.8	16.4
		GM-CSF	25	0.0	546.6	35.8	21.5	6.4	4.0	24.1	10.1	83.2
		IFN-γ	25	0.0	58.2	10.9	3.0	3.5	1.6	12.2	5.6	17.3
		TNF-α	25	0.0	213.8	8.7	8.5	0.0	0.0	0.0	0.0	25.9
	Affected	IL-2	29	0.0	422.8	33.3	14.3	13.8	7.0	30.9	14.8	64.6
		IL-6	29	3.9	13,610.2	497.2	468.4	14.1	10.3	31.0	18.3	1445.6
		IL-7	29	0.0	522.2	48.9	17.3	27.0	17.5	45.8	27.2	86.8
		IL-8	29	3304.7	64,303.7	22,711.1	2486.9	22,920.5	12,709.9	29,189.3	18,179.3	27,798.9
		IL-10	29	4.3	11,782.3	441.8	405.0	34.5	20.7	49.8	31.1	1257.8
		IL-15	29	0.0	778.3	84.2	27.0	44.1	31.1	71.9	44.6	145.6
		IL-18	29	7.0	647.6	57.2	21.7	21.5	17.6	60.1	28.3	104.1
		CCL2	29	970.9	134343.7	6800.1	4556.6	2200.6	1769.9	2692.6	2066.1	16041.4
		CXCL1	29	470.7	6409.5	1469.7	219.5	1271.6	769.8	1733.1	1101.3	1936.1
		CXCL10	29	0.0	33.1	9.8	1.2	9.0	5.8	12.6	7.6	12.3
		GM-CSF	29	0.0	491.8	42.5	16.6	15.7	11.5	44.1	20.1	78.5
		IFN-γ	29	1.8	42.6	10.8	1.9	7.7	2.9	13.9	7.6	14.7
		TNF-α	29	0.0	239.9	12.9	8.8	0.0	0.0	0.0	0.7	32.2

		Cytokine	*n*	Minimum	Maximum	Mean	s.e.m.	Median	Q1	Q3	95% CI of mean	
											Lower	Upper
Age group 3 (6 to <9 months)	Normal	IL-2	** **19	** **0.0	** **60.8	** **13.2	** **5.2	** **0.0	** **0.0	** **23.0	** **4.0	** **23.9
		IL-6	19	0.0	136.4	19.5	7.9	4.2	0.0	14.4	7.0	36.0
		IL-7	19	0.0	110.7	18.2	6.4	6.2	0.0	20.7	7.6	32.2
		IL-8	19	2454.6	20,794.5	8846.0	1336.7	6994.7	4209.1	10,680.7	6364.0	11,493.5
		IL-10	19	0.0	320.7	27.8	16.4	12.6	6.5	18.4	9.1	61.6
		IL-15	19	0.0	382.7	49.1	21.1	15.2	0.0	51.3	16.1	94.6
		IL-18	19	0.0	130.4	33.4	9.1	21.0	8.7	30.2	17.7	51.9
		CCL2	19	116.9	1831.6	279.1	87.2	189.2	158.8	216.4	175.2	460.1
		CXCL1	19	139.6	2038.2	536.6	98.3	446.1	291.9	696.6	376.8	749.6
		CXCL10	19	0.0	35.5	7.5	2.1	5.2	2.6	7.3	3.9	11.9
		GM-CSF	19	0.0	173.5	22.2	9.6	8.2	0.5	23.7	7.5	43.1
		IFN-γ	19	0.0	69.9	6.9	3.7	1.9	0.9	3.8	1.8	15.4
		TNF-α	19	0.0	24.2	1.4	1.3	0.0	0.0	0.0	0.0	4.1
	Affected	IL-2	20	0.0	56.1	15.0	3.8	9.3	0.0	26.0	8.0	22.7
		IL-6	20	0.0	166.9	25.0	8.4	12.1	7.0	23.1	12.0	43.8
		IL-7	20	8.5	75.8	38.5	5.2	31.2	20.0	60.2	28.9	48.5
		IL-8	20	1843.2	33,097.0	14,895.7	1712.9	12,992.7	9561.3	18,111.0	11,870.0	18,145.0
		IL-10	20	0.0	49.6	23.7	3.4	21.4	12.1	35.7	17.4	30.2
		IL-15	20	0.0	154.9	46.7	9.7	34.4	17.5	56.1	29.2	66.3
		IL-18	20	2.3	200.9	37.3	9.8	21.7	17.3	46.4	21.4	59.3
		CCL2	20	434.7	3593.5	1643.3	181.3	1729.9	984.7	2182.8	1290.9	2007.9
		CXCL1	20	414.8	2277.5	980.9	87.2	882.4	742.3	1121.7	829.8	1154.8
		CXCL10	20	0.0	79.7	9.4	3.9	5.5	0.0	9.9	3.6	17.8
		GM-CSF	20	0.0	163.3	36.2	7.9	22.9	14.9	51.1	22.8	53.6
		IFN-γ	20	0.0	30.1	6.1	1.6	3.6	1.8	7.3	3.4	9.4
		TNF-α	20	0.0	23.8	2.6	1.4	0.0	0.0	0.0	0.1	5.6

		Cytokine	*n*	Minimum	Maximum	Mean	s.e.m.	Median	Q1	Q3	95% CI of mean	
											Lower	Upper
Age group 4 (9 to <12 months)	Normal	IL-2	15	0.0	71.5	10.3	5.4	0.0	0.0	10.8	1.6	21.7
		IL-6	15	0.0	55.0	12.0	3.7	7.9	1.8	16.6	6.0	20.0
		IL-7	15	0.0	159.7	27.2	10.6	9.1	5.3	29.7	11.2	49.8
		IL-8	15	1889.6	13,174.4	5672.9	824.2	4929.0	3479.0	6237.3	4202.0	7310.9
		IL-10	15	0.0	34.6	8.9	3.0	1.5	0.0	15.7	3.7	14.8
		IL-15	15	0.0	185.0	45.1	16.9	0.0	0.0	82.1	16.3	80.9
		IL-18	15	0.0	311.9	42.4	20.4	9.9	3.3	51.6	13.6	86.1
		CCL2	15	132.2	471.6	207.6	25.1	173.5	144.6	215.8	166.6	259.9
		CXCL1	15	159.4	813.0	408.4	44.6	369.4	316.7	538.7	328.4	495.8
		CXCL10	15	1.6	34.3	11.0	2.7	6.1	3.5	18.6	6.3	16.1
		GM-CSF	15	0.0	198.9	27.3	13.5	2.0	0.0	39.7	7.2	56.5
		IFN-γ	15	0.0	13.0	2.3	0.9	0.9	0.4	2.0	0.9	4.1
		TNF-α	15	0.0	22.7	3.7	1.9	0.0	0.0	2.5	0.6	7.6
	Affected	IL-2	18	0.0	391.2	34.9	21.7	5.3	0.0	17.5	6.3	83.0
		IL-6	18	0.0	281.2	32.6	16.6	7.5	0.0	19.0	7.3	69.7
		IL-7	18	0.0	261.9	46.5	15.8	23.3	0.0	50.7	20.6	79.6
		IL-8	18	153.6	19,433.2	10,887.4	1327.1	10,894.7	6835.9	15,439.0	8313.2	13,431.7
		IL-10	18	0.0	33.1	13.3	2.3	13.0	5.0	20.7	8.9	17.7
		IL-15	18	0.0	688.8	71.6	38.5	20.6	0.0	46.6	17.7	156.2
		IL-18	18	3.3	262.6	43.4	18.2	14.1	6.6	35.1	14.6	83.0
		CCL2	18	11.7	1944.7	933.8	94.2	897.6	671.8	1203.0	749.3	1128.4
		CXCL1	18	0.0	1762.1	788.1	105.9	727.6	446.4	1099.1	585.4	1000.4
		CXCL10	18	0.0	36.0	11.3	2.5	11.1	4.4	12.2	6.9	16.0
		GM-CSF	18	0.0	198.4	34.0	12.8	11.3	5.8	29.3	13.1	62.4
		IFN-γ	18	0.0	25.3	3.9	1.4	2.7	1.4	3.4	1.7	6.9
		TNF-α	18	0.0	210.5	19.4	12.1	0.0	0.0	3.4	2.4	44.7

		Cytokine	*n*	Minimum	Maximum	Mean	s.e.m.	Median	Q1	Q3	95% CI of mean	
											Lower	Upper
Age group 5 (12 to <24 months)	Normal	IL-2	22	0.0	188.3	10.7	8.6	0.0	0.0	0.0	0.5	28.7
		IL-6	22	0.0	24.0	3.7	1.5	0.0	0.0	3.8	1.2	6.9
		IL-7	22	0.0	198.5	26.6	10.4	8.5	2.7	24.2	9.8	47.9
		IL-8	22	1590.3	14,072.0	5311.3	704.8	5074.2	2634.2	6279.8	4040.0	6709.2
		IL-10	22	0.0	45.8	7.1	2.4	1.1	0.0	8.9	3.0	12.3
		IL-15	22	0.0	134.8	16.8	7.3	0.0	0.0	28.5	5.1	32.2
		IL-18	22	0.0	105.6	21.9	6.5	8.2	3.0	28.9	10.1	35.4
		CCL2	22	79.4	242.7	151.4	9.9	142.6	116.9	193.0	133.4	169.9
		CXCL1	22	23.8	1103.2	425.8	64.6	417.5	167.3	555.3	308.5	547.1
		CXCL10	22	0.0	70.6	10.8	3.8	3.5	0.0	12.4	4.5	18.7
		GM-CSF	22	0.0	53.0	8.3	2.9	1.9	0.0	11.1	3.5	14.0
		IFN-γ	22	0.0	13.0	2.2	0.7	0.9	0.0	2.9	1.0	3.5
		TNF-α	22	0.0	0.0	0.0	0.0	0.0	0.0	0.0	0.0	0.0
	Affected	IL-2	22	0.0	127.9	21.3	7.3	6.4	0.0	32.6	9.0	36.6
		IL-6	22	0.0	161.1	24.3	8.0	8.8	7.1	19.0	11.4	42.1
		IL-7	22	0.0	265.0	34.2	11.9	18.1	7.6	35.0	17.1	61.1
		IL-8	22	4443.9	35,775.9	11,630.9	1549.0	10,687.3	6000.6	13,705.2	8986.7	14,707.9
		IL-10	22	0.0	82.6	17.2	3.8	12.4	5.9	21.1	11.0	25.5
		IL-15	22	0.0	416.7	43.2	18.6	16.3	0.0	49.6	17.4	84.1
		IL-18	22	0.0	387.7	44.4	18.3	18.5	10.5	27.7	17.3	87.0
		CCL2	22	158.0	1101.9	518.7	45.3	509.3	407.9	592.5	438.6	607.5
		CXCL1	22	206.0	3645.3	781.5	151.4	649.4	404.7	953.2	548.5	1111.6
		CXCL10	22	0.0	61.1	10.8	2.8	7.3	3.9	12.0	6.3	16.7
		GM-CSF	22	0.0	293.5	35.5	13.3	18.2	5.0	43.4	16.0	64.8
		IFN-γ	22	0.0	18.1	3.2	1.0	1.2	0.3	5.2	1.5	5.2
		TNF-α	22	0.0	78.9	6.6	3.9	0.0	0.0	0.0	0.3	14.9

		Cytokine	*n*	Minimum	Maximum	Mean	s.e.m.	Median	Q1	Q3	95% CI of mean	
											Lower	Upper
Age group 6 (24 to <46 months)	Normal	IL-2	15	0.0	38.7	8.3	4.0	0.0	0.0	7.8	1.4	16.4
		IL-6	15	0.0	44.1	12.0	4.0	5.5	0.0	16.8	5.2	20.5
		IL-7	15	0.0	48.2	13.1	3.6	8.9	1.8	17.2	6.9	20.5
		IL-8	15	0.0	10,949.3	4338.4	752.3	3587.6	2369.5	5902.9	2957.2	5812.0
		IL-10	15	0.0	32.5	8.9	2.8	5.0	0.0	13.3	4.1	14.4
		IL-15	15	0.0	56.9	10.7	5.0	0.0	0.0	22.5	2.1	20.6
		IL-18	15	0.0	54.0	13.0	4.2	6.0	4.3	10.6	6.0	21.6
		CCL2	15	113.2	285.3	165.1	10.8	154.4	141.4	180.0	146.6	187.7
		CXCL1	15	111.7	650.5	283.1	41.0	216.9	173.8	380.4	210.1	366.7
		CXCL10	15	0.0	58.4	16.1	4.0	12.6	5.8	24.1	9.3	24.1
		GM-CSF	15	0.0	42.6	8.5	3.4	2.9	0.0	10.7	2.9	15.4
		IFN-γ	15	0.0	13.8	2.1	0.9	1.5	0.3	1.7	0.9	4.0
		TNF-α	15	0.0	0.0	0.0	0.0	0.0	0.0	0.0	0.0	0.0
	Affected	IL-2	22	0.0	392.5	26.1	17.7	0.0	0.0	17.4	4.7	64.0
		IL-6	22	0.0	1744.6	94.6	78.9	5.7	0.0	10.6	6.2	259.8
		IL-7	22	0.0	961.6	52.5	43.4	4.0	0.0	12.9	5.6	141.4
		IL-8	22	1312.0	22,442.3	8906.2	1169.9	7004.2	5777.2	11,157.9	6887.8	11,235.2
		IL-10	22	0.0	221.6	16.6	9.9	6.1	0.0	12.6	4.7	37.8
		IL-15	22	0.0	1936.6	115.3	88.5	0.0	0.0	20.6	7.3	307.1
		IL-18	22	0.0	1203.6	66.0	54.3	7.1	4.2	12.0	6.8	177.5
		CCL2	22	145.9	1741.9	564.1	76.4	466.0	355.9	637.4	434.2	727.0
		CXCL1	22	158.5	1119.8	548.5	61.0	570.3	327.5	693.4	435.3	665.6
		CXCL10	22	0.0	97.3	15.7	4.7	9.3	3.0	14.7	8.2	25.9
		GM-CSF	22	0.0	897.1	49.3	40.5	3.5	0.0	11.2	4.3	132.0
		IFN-γ	22	0.0	54.2	4.7	2.5	0.2	0.0	4.8	1.2	10.4
		TNF-α	22	0.0	181.2	9.2	8.2	0.0	0.0	0.0	0.0	26.4

CI, confidence interval; Q1, 25th percentile quartile; Q3, 75th percentile quartile.

Some dogs were measured multiple times in the same age group. These data were considered as repeated measurements in the linear mixed-effects model (Fig. 3). However, only the median values from all such repeated measurements were used to calculate reference values for each age group to avoid potential bias (Table 1). Therefore, no dogs appeared more than once in the same age group when calculating the reference values presented in Table 1.

No statistically significant genotype effects were found in any age group for IFN-γ (Fig. 3). For the remaining 12 analytes, statistical significance was detected in at least one age group. Notably, nine analytes (IL-2, IL-6, IL-7, IL-8, IL-10, IL-15, IL-18, CCL2 and CXCL1) were significantly higher in affected dogs than in normal dogs in age group 2. Although cytokine/chemokine levels were higher in affected dogs than in normal dogs in most cases, CXCL10 was the only exception and had significantly higher concentrations in normal dogs younger than 3 months than in age-matched affected dogs (Fig. 3).

Of notice, CCL2 was significantly elevated in affected dogs in all age groups. In normal dogs, the median values of CCL2 were 310.92, 261.15, 189.22, 173.48, 142.55 and 154.40 pg/ml in age groups 1, 2, 3, 4, 5 and 6, respectively. In affected dogs, the median values of CCL2 were 893.38, 2200.59, 1729.90, 897.61, 509.28 and 465.95 pg/ml in age groups 1, 2, 3, 4, 5 and 6, respectively (Table 1; Table S3). The fold differences in the median values were most pronounced in age groups 2 and 3 (8- to 9-fold), followed by age group 4 (5-fold) (Table S3). The remaining age groups showed a ∼3-fold difference.

### Male and female dogs show similar cytokine/chemokine levels

Both male and female dogs were included in the study. To determine whether the cytokine/chemokine levels were influenced by sex, we compared values obtained from male and female dogs. The linear mixed-effects model analysis indicated that only CCL2 showed a significant difference between male and female dogs (Table S4). However, when analyzed in each age group, the significant sex effect on CCL2 was confirmed only in age group 3 (6 to <9 months) (Table S4).

### Multiple cytokines/chemokines show age-associated reduction

Next, we examined the age effect. The linear mixed-effects model, with age as a fixed factor, revealed a statistically significant age effect in 12 of the 13 cytokines/chemokines, except for TNF-α. The estimated age coefficients were negative for all but CXCL10, indicating a reduction over time (Table S5). A statistically significant age effect was found for CXCL10, with a positive estimated age coefficient (Table S5).

### CCL2 distinguishes normal and affected dogs

We applied principal component analysis (PCA) to investigate whether combinations of some cytokines can result in a smaller number of features to explain the variations in the entire dataset (Fig. S2, Table S6). Unfortunately, principal component (PC)1 only explained 40% of the variance in the dataset. The first six components together only explained 79.8% of the variations in the dataset (Fig. S2B). In addition, combined use of PC1 and PC2 failed to satisfactorily separate normal and affected dogs (Fig. S2C). Collectively, the PCA is not effective for our dataset.

We used the minimum redundancy maximum relevance (MRMR) algorithm to rank the cytokines/chemokines for discriminating sera from normal and affected dogs. This analysis revealed CCL2 as the best predictor to classify normal and affected dogs (MRMR score: 0.33) (Table S7). The MRMR scores were <0.08 for the remaining cytokines/chemokines. Because age has a significant effect on most cytokines/chemokines, we included age as a predictor in machine learning. A cubic support vector machine (SVM) was trained using CCL2 (log transformed) and age (in months) as two predictors. The model achieved an accuracy of 89.3% in identifying serum samples from normal and affected dogs, with 91.2% sensitivity (true positive rate) and 86.8% specificity (true negative rate). IL-8 and CXCL10 had the next two highest MRMR scores (0.076 and 0.072, respectively). Including IL-8 and CXCL10 in the SVM model only resulted in slight changes in accuracy (90.9%), sensitivity (90.8%) and specificity (91.0%). These results confirmed that CCL2 was the best feature to distinguish normal and affected dogs.

### Most cytokines/chemokines yield similar readings in fresh and frozen serum

**Fig. 4.**
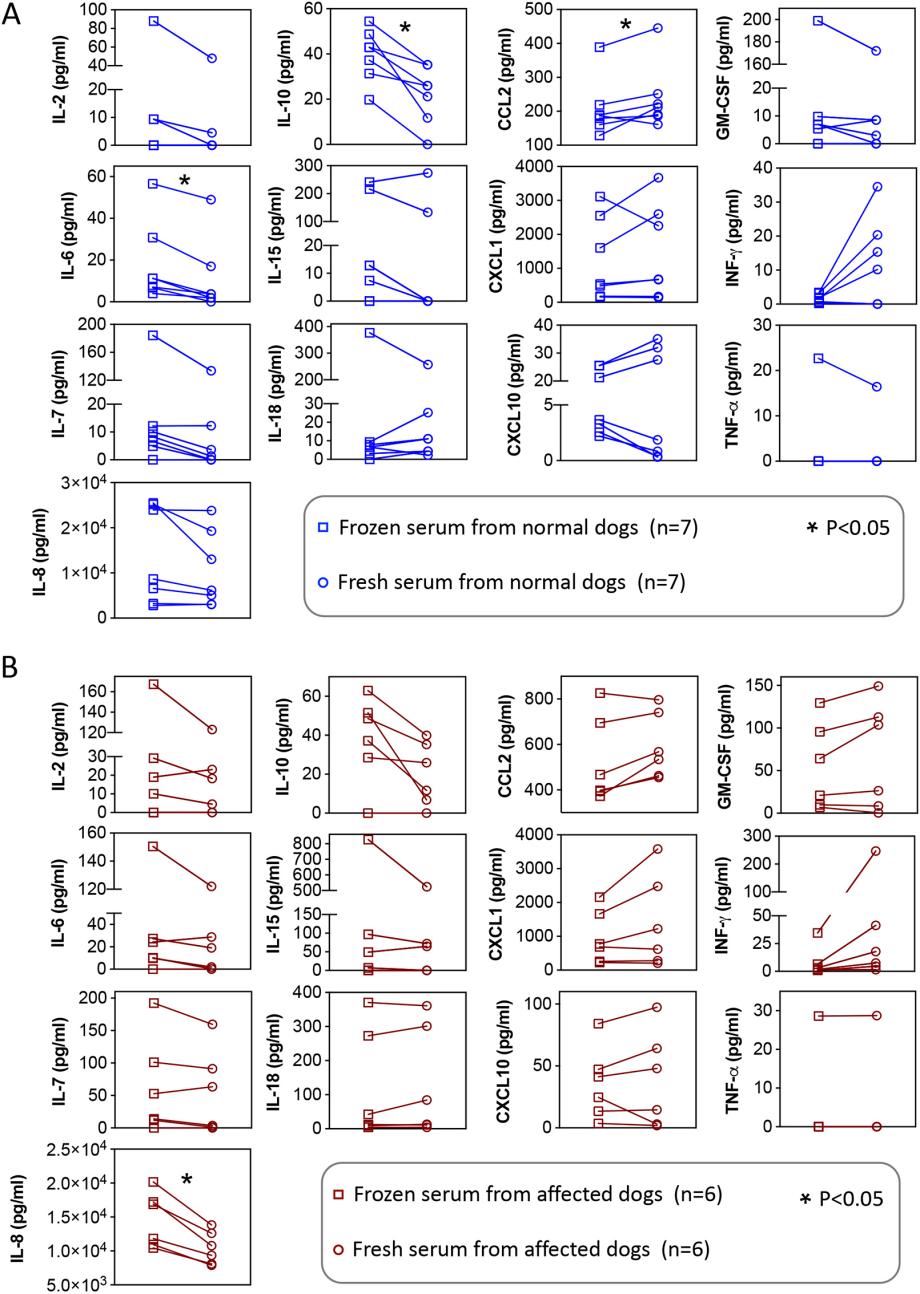
**Comparison of cytokine/chemokine levels in fresh and frozen serum from the same dogs.** Serum samples were analyzed immediately after collection or stored at −80°C and thawed once. Data were analyzed using the Wilcoxon signed-rank test. (A) Paired comparison of serum samples collected from normal dogs. (B) Paired comparison of serum samples collected from affected dogs. *P<0.05.

In this study, we used curated serum samples. These samples were stored in a −80°C freezer. To determine whether storage at −80°C affected results, we compared the cytokine/chemokine levels in freshly collected and frozen serum from the same dog in 13 dogs (seven normal and six affected dogs) (Fig. 4). No statistical significance was detected between fresh and frozen serum for most cytokines/chemokines. The only exceptions were IL-6, IL-10 and CCL2 in normal dogs and IL-8 in affected dogs. The mean values of IL-6 and IL-10 were 1.65-fold and 1.79-fold higher, respectively, in frozen serum than in fresh serum in normal dogs. However, the mean value of CCL2 was 1.15-fold higher in fresh serum than in frozen serum in normal dogs. In affected dogs, the mean value of IL-8 was 1.40-fold higher in frozen serum than in fresh serum.

### Most cytokines/chemokines show higher readings in serum than in plasma

**Fig. 5.**
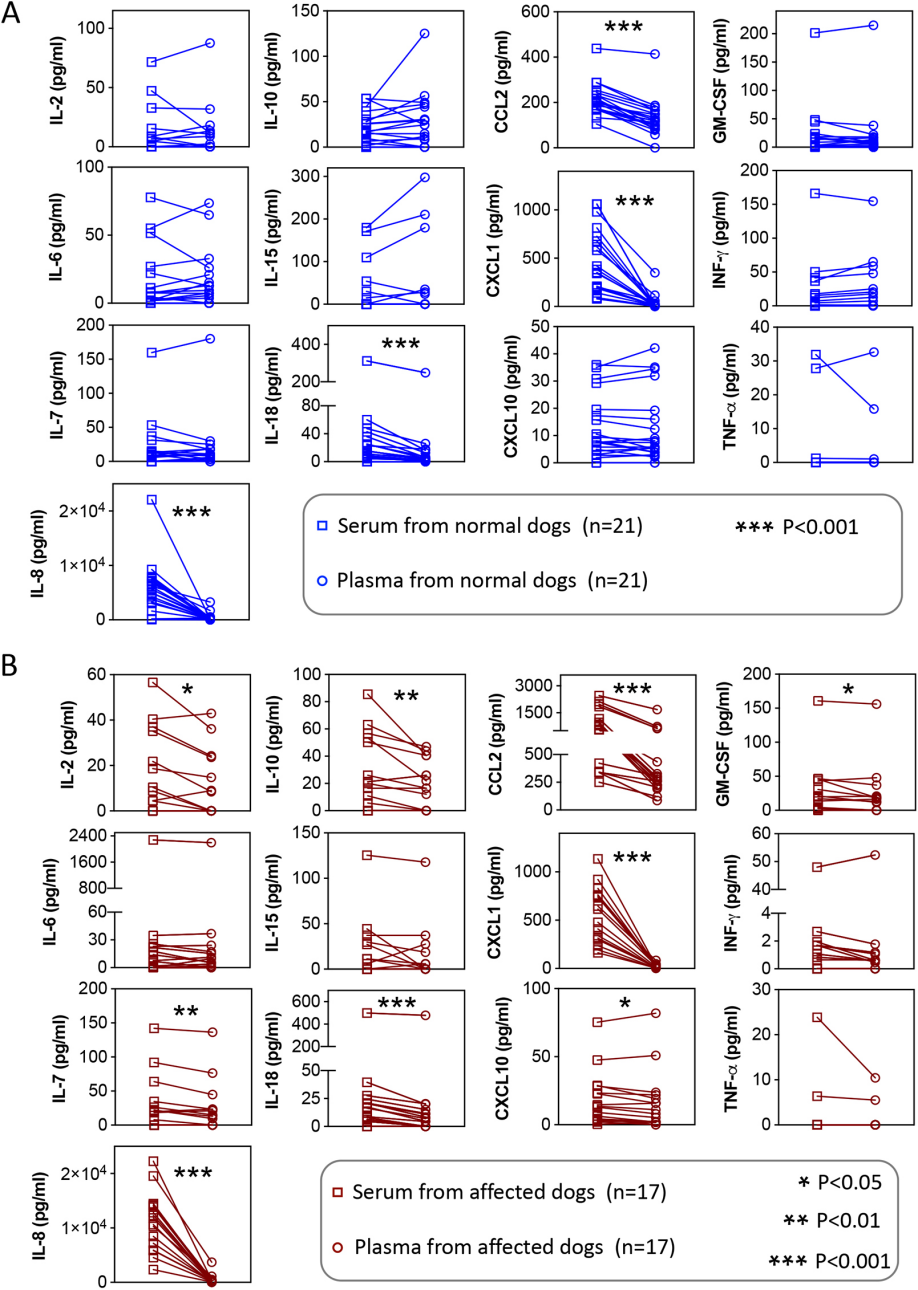
**Comparison of cytokine/chemokine levels in frozen serum and plasma from the same dogs.** Serum and plasma samples were frozen at −80°C and thawed once. Data were analyzed using the Wilcoxon signed-rank test. (A) Paired comparison of samples collected from normal dogs. (B) Paired comparison of samples collected from affected dogs. *P<0.05; **P<0.01; ***P<0.001.

Serum samples were used in the cytokine/chemokine analysis. Plasma samples have been used to quantify circulating cytokine/chemokine levels in the literature (Gaertner and Massberg, 2016; Henno et al., 2017; Rosenberg-Hasson et al., 2014). To determine whether the results obtained from serum were equivalent to those obtained from plasma, we compared the cytokine/chemokine levels in frozen serum and plasma from the same dog in 38 dogs (21 normal dogs and 17 affected dogs) (Fig. 5). No significant difference was detected in four analytes (IL-6, IL-15, IFN-γ and TNF-α). Statistical significance was detected in normal and/or affected dogs for the remaining nine analytes. In normal dogs, serum levels of IL-8, IL-18, CCL2 and CXCL1 were significantly higher than plasma levels (Fig. 5A). The differences in the mean values were moderate for IL-18 and CCL2 (1.83-fold and 1.62-fold, respectively) but quite substantial for IL-8 and CXCL1 (14.08-fold and 13.10-fold, respectively). In affected dogs, serum levels of IL-2, IL-7, IL-8, IL-10, IL-18, CCL2, CXCL1, CXCL10 and GM-CSF were significantly higher than plasma levels (Fig. 5B). The differences in the mean values were moderate for most cytokines/chemokines (1.49-fold, 1.24-fold, 1.48-fold, 1.25-fold, 2.34-fold, 1.14-fold and 2.12-fold for IL-2, IL-7, IL-10, IL-18, CCL2, CXCL10 and GM-CSF, respectively). As observed in normal dogs, substantial differences were observed for IL-8 and CXCL1 in affected dogs. Mean serum levels of IL-8 and CXCL1 were 26.04-fold and 25.01-fold higher than mean plasma levels, respectively.

## DISCUSSION

In this study, we compared the circulatory levels of 13 cytokines/chemokines in dystrophin-deficient and normal control canines. Ten cytokines/chemokines (IL-2, IL-6, IL-7, IL-8, IL-10, IL-15, IL-18, CCL2, CXCL1 and GM-CSF) were significantly elevated in affected dogs. Three cytokines/chemokines (CXCL10, IFN-γ and TNF-α) showed minimal differences. CCL2 was consistently elevated in all affected dogs irrespective of age, with the largest difference appearing in 3- to <9-month-old dogs. Combined use of CCL2 and age effectively classified normal and affected dogs with ∼90% accuracy.

Cytokines are small secreted signaling proteins that mediate cellular communications and interactions. Chemokines are low-molecular mass (8-13 kDa) chemotactic cytokines that recruit immune cells to the site of injury. Cytokines/chemokines play important roles in the inflammatory response. Cytokine/chemokine dysregulation is an inherent feature of DMD (De Paepe and De Bleecker, 2013; Evans et al., 2009; Rosenberg et al., 2015). Multiple cytokines/chemokines are upregulated in the muscle and serum of patients with DMD. Among 13 cytokines/chemokines investigated, six (IL-2, IL-6, IL-8, CCL2, CXCL1 and TNF-α) have been shown to be elevated in serum and/or muscle of patients with DMD. Specifically, IL-2 is elevated in serum of patients with DMD (Chahbouni et al., 2010). IL-6 (Chahbouni et al., 2010; Messina et al., 2011; Rufo et al., 2011), CCL2 (De Paepe et al., 2012; Hathout et al., 2019; Ogundele et al., 2021; Pescatori et al., 2007), CXCL10 (Ogundele et al., 2021) and TNF-α (Abdel-Salam et al., 2009; Chahbouni et al., 2010; Comim et al., 2015; Messina et al., 2011; Tews and Goebel, 1996) are increased in both muscle and serum in patients with DMD. IL-8 and CXCL1 are increased in muscle in patients with DMD (De Paepe et al., 2012).

Besides human patients, numerous studies have documented cytokine/chemokine elevation in mouse models of DMD. However, only a few examined cytokine/chemokine changes in the canine DMD models. Nakamura et al. (2013) found that IL-6 and IL-8 are significantly upregulated in the diaphragm of affected dogs. We observed a significant elevation of *IL2*, *IL15*, *IL18*, *TNFA* and *IFNG* transcripts in affected dog muscle in the context of the Cas9-induced cellular immune response (Hakim et al., 2021). Recently, Riddell et al. (2022) examined the serum levels of the same 13 cytokines/chemokines in the DE50-MD dog model using the same canine-specific Luminex assay. In the DE50-MD model, a point mutation in intron 50 results in an exon 50-deleted transcript and frameshift (Walmsley et al., 2010). This model has been bred to the Beagle background. In the study by Riddell et al. (2022), 14 affected male dogs and 11 age- and sex-matched normal littermates were tested at 3 (12 affected and 11 normal), 6 (12 affected and 11 normal), 9 (seven affected and 11 normal), 12 (seven affected and 11 normal), 15 (six affected and nine normal) and 18 (five affected and nine normal) months of age. The authors found that (1) 11 cytokines/chemokines (IL-2, IL-6, IL-7, IL-8, IL-10, IL-15, IL-18, CCL2, CXCL1, GM-CSF and TNF-α) were significantly increased in affected dogs at one or more time points; (2) most differences occurred at 9 months of age (ten cytokines/chemokines were significantly elevated); (3) only CCL2 was significantly elevated at all time points; and (4) no difference was detected for CXCL10 and IFN-γ (Riddell et al., 2022).

Because human patients with DMD are genetically diverse, and both males and females can be affected (Duan et al., 2021), we performed our study in mixed-breed dogs of both sexes. Unlike DE50-MD dogs, affected dogs in our study carry one (all males and some females) or two (some females) of three distinctive mutations, including point mutation in intron 6 and long interspersed nuclear element-1 insertion in intron 13 or 19 (McGreevy et al., 2015). To gain a comprehensive understanding of the natural history, we included 428 serum samples collected from birth to 45.2 months from 90 affected dogs and 73 normal dogs (Fig. 1).

Linear mixed-effects model analysis revealed significant elevation of ten cytokines/chemokines (IL-2, IL-6, IL-7, IL-8, IL-10, IL-15, IL-18, CCL2, CXCL1 and GM-CSF) in affected dogs, but no difference was found between normal and affected dogs for CXCL10, TNF-α and IFN-γ (Fig. 2). Subsequent age group-based analysis not only detected significant elevation of the same ten cytokines/chemokines in affected dogs in two or more age groups but also found a significant reduction in CXCL10 in <3-month-old affected dogs and a significant increase in TNF-α in 12- to <24-month-old affected dogs.

Our findings are, in general, consistent with the results of Riddell et al. (2022). However, there are some differences. For example, Riddell et al. (2022) showed that most elevations occurred at 9 months of age in affected dogs. We found that nine cytokines/chemokines were significantly elevated in the 3- to <6-month-old age group in affected dogs, while fewer cytokine/chemokine elevations were detected in 6- to <9-month-old (six cytokines/chemokines) and 9- to<12-month-old (three cytokines/chemokines) age groups (Fig. 3). Dystrophin-deficient canines are minimally affected in the first 3 months of their life (Kornegay, 2017; McGreevy et al., 2015). They begin to show clinical disease and develop marked muscle degeneration, necrosis and inflammation between 3 and 6 months of age. Their clinical presentations tend to stabilize afterward (Kornegay, 2017; McGreevy et al., 2015). Our results align with the clinical course of affected dogs, suggesting that muscle damage and inflammation are likely to be the direct cause of increased circulatory cytokines/chemokines during this period. It is unclear why the kinetic profiles of cytokine/chemokine elevation differ between the two studies [in our study, it occurs between 3 and 6 months; in Riddell et al. (2022), it occurs at 9 months]. We speculate that these differences relate to the sample size (ours is larger) and age group definition [ours is continuous, that of Riddell et al. (2022) is not]. Further, the location of *DMD* gene mutation [ours is near the 5′ end, that of Riddell et al. (2022) is near the 3′ end] and genetic background [ours is mixed, that of Riddell et al. (2022) is pure] could also have contributed to the observed differences.

Riddell et al. (2022) showed that TNF-α was significantly increased in affected dogs between 6 and 15 months of age. However, we only detected significant elevation in 12- to <24-month-old affected dogs (Fig. 3). In the study by Riddell et al. (2022), TNF-α was below the detection limit in 80.65% of normal (50 of 62) and 28.00% of affected (14 of 50) dog samples. The authors replaced these with the lowest value within the dataset in the analysis. In our study, TNF-α was below the detection limit in 90.48% of normal and 84.52% of affected dog samples. We replaced these with 0.1 pg/ml, rather than the lowest value of the dataset (1.64 pg/ml) in the analysis (Table S1). Likely, the differences in data distribution and handling, sample size and age group definition created the discrepancy.

An unexpected finding is the significant reduction in CXCL10 in <3-month-old affected dogs (Fig. 3). It has been shown that CXCL10 is significantly increased in the serum of ≥4-year-old patients with DMD (Hathout et al., 2015; Ogundele et al., 2021). However, no significant difference was detected in ≥3-month-old dogs in our study and in Riddell et al. (2022) (Fig. 3). Interestingly, a previous study in the *mdx* mouse model also failed to detect a significant increase in serum CXCL10 (Ogundele et al., 2021). The serum CXCL10 level obtained from animal studies appears to be unsuitable for predicting changes in patients with DMD. Hence, our unexpected observation might have few translational implications.

The most consistent finding between our study and that of Riddell et al. (2022) is CCL2. It was significantly elevated at all ages in both studies. Further, machine learning showed that CCL2 best distinguished normal and affected dogs (Table S7). CCL2 is a proinflammatory chemokine produced by a variety of immune cells such as neutrophils and macrophages (Deshmane et al., 2009). It can also be produced by injured muscle (Lu et al., 2011). The primary function of CCL2 is to recruit myeloid cells (especially macrophages) to the site of damage. CCL2 also plays a critical role in muscle regeneration (Shireman et al., 2007). CCL2 is elevated in many diseases, such as cancer, rheumatoid arthritis, diabetes and cardiovascular diseases (Deshmane et al., 2009). In the context of DMD, CCL2 expression is not only increased in muscle and serum of human patients and affected dogs, but is also significantly increased in *mdx* muscle (Bronisz-Budzynska et al., 2019; Demoule et al., 2005; Hyzewicz et al., 2017; Mojumdar et al., 2014; Porter et al., 2003) and *mdx* serum (Kranig et al., 2019; Ogundele et al., 2021). Importantly, CCL2 elevation can be detected before the onset of clinical symptoms in human patients (Pescatori et al., 2007), before massive muscle necrosis in *mdx* mice (Porter et al., 2003) and before clinical disease in affected dogs (Fig. 3) (Riddell et al., 2022). Collectively, CCL2 could represent an excellent cross-species cross-disease course biomarker for translating findings from animal models to patients with DMD. Indeed, CCL2 has been used as a biomarker in preclinical studies (Huynh et al., 2013; Hyzewicz et al., 2017; Merckx et al., 2022; Mojumdar et al., 2014) and clinical trials (NCT02439216, NCT02760264) (Chahbouni et al., 2010).

In this study, we used curated frozen serum samples to evaluate the differences between normal and affected dogs. Unlike fresh serum, frozen serum has a unique advantage. Multiple frozen samples collected at different time points can be processed simultaneously. However, it has been suggested that the freeze–thaw process can influence the measured cytokine/chemokine concentrations (Simpson et al., 2020). To determine whether storage at −80°C skewed the outcome, we compared cytokine/chemokine levels in fresh and frozen serum from the same dogs. Consistent values were obtained for most cytokines/chemokines, suggesting that the use of frozen serum did not substantially alter our findings (Fig. 4).

Serum is prepared after the blood is clotted. It has been shown that some cytokines/chemokines have higher levels in serum than in plasma because the coagulation process induces cytokine/chemokine release from blood leukocytes and platelets (Gaertner and Massberg, 2016; Henno et al., 2017; Rosenberg-Hasson et al., 2014). To determine whether the use of serum instead of plasma affected our results, we compared cytokine/chemokine levels in serum and plasma from the same dogs (Fig. 5). Consistent with the literature, we found that multiple cytokines/chemokines were significantly increased in serum. Serum values of nine cytokines/chemokines (IL-2, IL-7, IL-8, IL-10, IL-18, CCL2, CXCL1, CXCL10 and GM-CSF) were significantly elevated compared to their plasma values in affected dogs. Only four of these nine cytokines/chemokines (IL-8, IL-18, CCL2 and CXCL1) showed significantly higher serum values than plasma values in normal dogs. Of these four, three (IL-8, CCL2 and CXCL1) showed a larger magnitude of elevation in affected dogs than that in normal dogs (26-fold, 2.34-fold and 25-fold in affected dogs for IL-8, CCL2 and CXCL1, respectively; 14-fold, 1.62-fold and 13-fold in normal dogs for IL-8, CCL2 and CXCL1, respectively). Our results suggest that more cytokines/chemokines were released during coagulation in affected dogs than in normal dogs. Future studies are needed to determine whether this is a consequence of muscle disease. For example, DMD could have increased leukocyte and/or platelet numbers in the blood. Alternatively, DMD could have made leukocytes and/or platelets more susceptible to coagulation-induced cytokine/chemokine release. Given the more pronounced elevation of cytokines/chemokines in the serum of affected dogs, the difference between normal and affected dogs could be smaller if circulatory cytokines/chemokines were measured in plasma.

To determine whether the difference between serum and plasma compromised the robustness of CCL2 as a biomarker to distinguish affected dogs, we calculated the hypothetical plasma values based on the mean serum-to-plasma ratio in normal (1.62) and affected (2.34) dogs (Table S3). We then reanalyzed the data using the linear mixed-effects model. As expected, the differences between affected and normal dogs were reduced in all age groups when hypothetical plasma values were used in the analysis (Table S3). However, the CCL2 levels in affected dogs remained significantly higher than those in normal dogs in age groups 2 to 6 (Fig. S3).

In summary, in this study, we confirmed and extended the findings of Riddell et al. (2022). Together, the results suggest that CCL2 is a robust circulatory cytokine/chemokine biomarker in the canine model of DMD.

## MATERIALS AND METHODS

### Experimental animals

All animal experiments were approved by the Animal Care and Use Committee of the University of Missouri and performed in accordance with National Institutes of Health guidelines. All animal experiments were conducted at the University of Missouri. All experimental dogs were on a mixed genetic background of the Golden Retriever, Labrador Retriever, Beagle, Spaniel and Welsh Corgi, and were generated in-house by artificial insemination. The genotype was determined by polymerase chain reaction (Fine et al., 2011; Hakim et al., 2021; Smith et al., 2011). All experimental dogs were housed in a specific animal care facility and kept under a 12 h light/12 h dark cycle with *ad libitum* access to clean drinking water. Normal dogs were fed with dry Laboratory Canine Diet 5006 (LabDiet, St Louis, MO, USA). Affected dogs were fed with wet Purina Pro Plan Puppy food or a mixture of dry and wet Purina Pro Plan Puppy food (Nestle Purina PetCare Company, St Louis, MO, USA) as instructed by the veterinarian. Affected dogs were housed in a raised platform kennel, whereas normal dogs were housed in a regular floor kennel. Depending on the age and size, two or more dogs were housed together to promote socialization. Toys were allowed in the kennel with dogs for activity enrichment. Dogs were monitored daily by the caregivers for overall health condition and activity. A complete physical examination was performed by the veterinarian from the Office of Animal Research at the University of Missouri for any unusual changes in behavior, activity, food and water consumption, and clinical symptoms. The body weights of the dogs were measured periodically to monitor growth and body condition (Fig. S4).

### Sample size

Sera from 163 dogs (73 normal and 90 affected) were collected to study muscle disease-, age- and sex-associated changes in cytokine/chemokine concentrations in the circulation. Sera from 13 dogs (seven normal and six affected) were collected to determine whether the freeze–thaw process affected cytokine/chemokine levels in the blood. Sera and plasma from 38 dogs (21 normal and 17 affected) were collected to determine whether they yielded similar cytokine/chemokine levels.

### Serum and plasma collection

Blood was collected from the jugular vein, cephalic vein or saphenous vein according to our standard operating protocol (Hakim et al., 2023). Serum was collected in a red-top tube with a silicone-coated interior (Becton Dickinson, Franklin Lakes, NJ, USA; 366668). The blood sample was allowed to clot fully at room temperature (usually in 10-15 min) and then centrifuged at 3050 ***g*** for 5 min at room temperature in an Eppendorf benchtop centrifuge (Millipore Sigma, St Louis, MO, USA; model number 5810R, rotor number A-4-62). Plasma was collected in a lavender-top spray-coated K_2_EDTA hematology tube (Becton Dickinson; 366643) and then centrifuged at 3050 ***g*** for 15 min at room temperature in an Eppendorf benchtop centrifuge (Millipore Sigma; model number 5810R, rotor number A-4-62). The serum was either directly used in the multiplex Luminex assay or frozen as aliquots at −80°C until use. Plasma was frozen as aliquots at −80°C until use. To quantify cytokine/chemokine levels in a frozen sample, the sample was thawed at room temperature and then centrifuged at 3655 ***g*** for 5 min at room temperature in an Eppendorf benchtop centrifuge (Millipore Sigma; model number 5417C, rotor number F45-30-11). The supernatant was used in the multiplex Luminex assay.

### Circulatory cytokine and chemokine quantification

A total of 13 cytokines and chemokines in the blood were quantified using the canine-specific multiplex Luminex kit according to the manufacturer's instructions (Millipore Sigma, Burlington, MA, USA; Milliplex MAP canine cytokine/chemokine magnetic bead panel, CCYTMG-90 K-PX13) (Hakim et al., 2023). All samples, standards and quality controls were analyzed in duplicate in the MAGPIX reader (Luminex, Austin, TX, USA) using the Luminex XPONENT software (Version 4.2). Data were analyzed using the Belysa software (Version 1.0.19; Millipore Sigma). Cytokine measurements with a mean fluorescence intensity (MFI) lower than or equal to the mean plus one standard deviation of background MFI were assigned as zero (below the detection limit of the assay).

### Statistical analysis

Data are presented as median±interquartile range, mean±s.d. and 95% CI. Outliers were defined as values beyond the fifth and 95th percentiles. Because cytokine measurements showed a large data range, they were visualized in logarithmic scales. For statistical analyses, we applied logarithm transformation as log(C+0.1), where C is the raw concentration measurement, and 0.1 was added to account for zero values. Statistical analysis was performed using the Statistics and Machine Learning Toolbox (Version 24.1, MATLAB 2024a; MathWorks, Natick, MA). The linear mixed-effects model was used to determine the statistical differences between normal and affected dogs, and to assess whether cytokine/chemokine concentrations changed significantly with age and sex. A random slope was incorporated to account for variations in individual dogs. To test whether sample storage (fresh versus frozen) and sample type (serum versus plasma) yielded significantly different measurement results, the Wilcoxon signed-rank test was used because most data did not follow normal distributions by the Anderson–Darling test. PCA was used to investigate whether combinations of certain cytokines/chemokines could reduce the number of variables needed to explain the original variability. We weighted PCA using the inverse variance of each measure to account for the large data range among different cytokine/chemokine measures. The MRMR algorithm was used for feature selection to identify the most informative and least redundant cytokines and chemokines for distinguishing between samples from normal and affected dogs. These selected features were then used to train a quadratic SVM model to classify the genotype of serum samples. A 10-fold cross-validation was employed for model training and validation. *P*<0.05 was considered statistically significant in all statistical analyses.

## Supplementary Material

10.1242/dmm.052137_sup1 Supplementary information
