# Supplementary material for: Circulatory CCL2 distinguishes Duchenne muscular dystrophy dogs
Source: Dis Model Mech. 2025 Mar 14;18(3):dmm052137. doi: 10.1242/dmm.052137 (PMC12140648; doi:10.1242/dmm.052137)
Supplement: Supplementary information [file dmm-18-052137-s1.pdf]

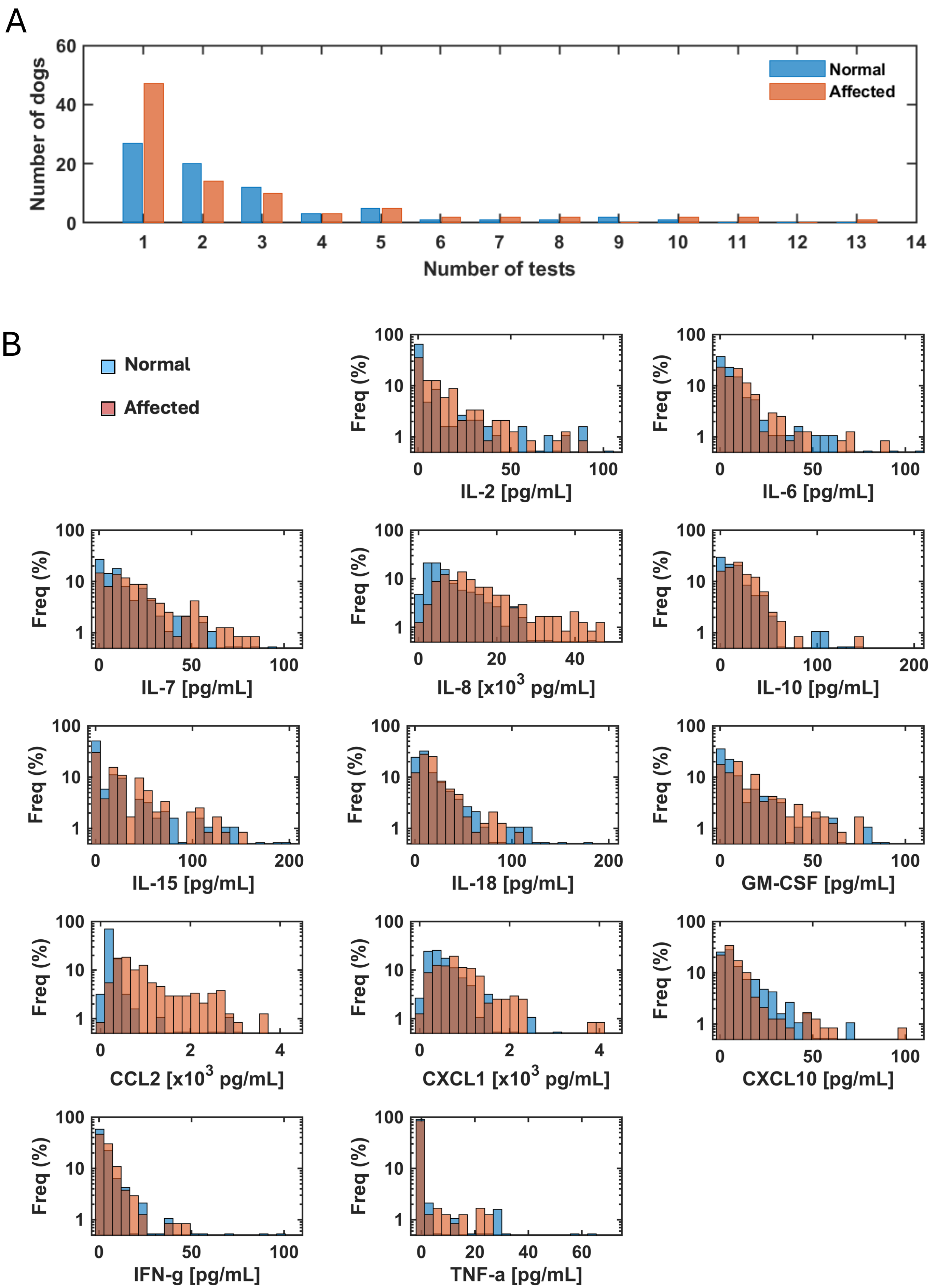

Disease Models & Mechanisms • Supplementary information

Fig. S1. Overview of the cytokine/chemokine data. A, Distribution of the frequency of sample collection in study dogs. **B,** Distribution of cytokine/chemokine concentrations. The frequency represents the percentage of the samples with a cytokine/chemokine concentration in the indicated range.

A

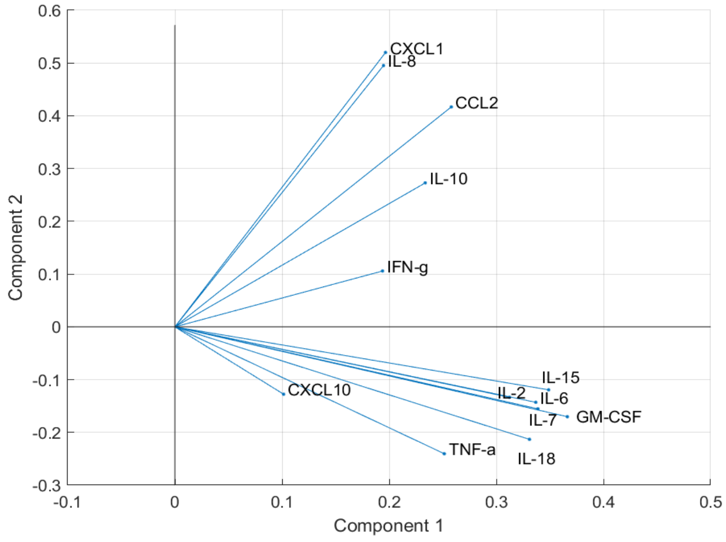
CXCL1
IL-8
CCL2
IL-10
IFN-g
IL-15
IL-2
IL-6
IL-7
GM-CSF
CXCL10
TNF-a
IL-18
Component 2
Component 1

B

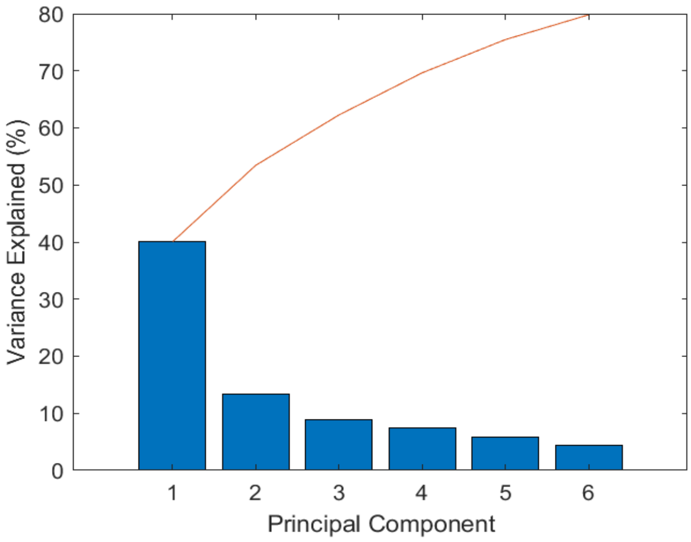
Variance Explained (%)
Principal Component

C

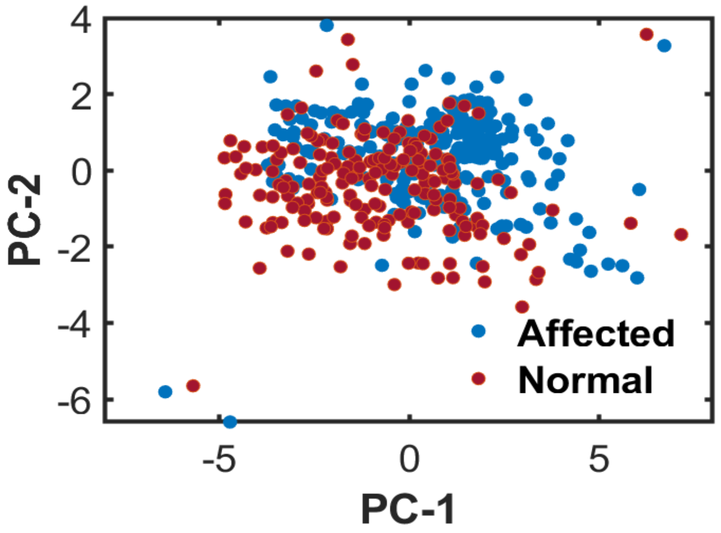
PC-2
PC-1
Affected
Normal

Fig. S2. Principal component (PC) analysis. A, Biplot presentation of the relative contribution of each cytokine/chemokine to principal components 1 and 2. **B,** Percentage of variance explained by each principal component. **C,** Scatter plot presentation of PC-1 and PC-2 from normal and affected dogs.

Disease Models & Mechanisms • Supplementary information

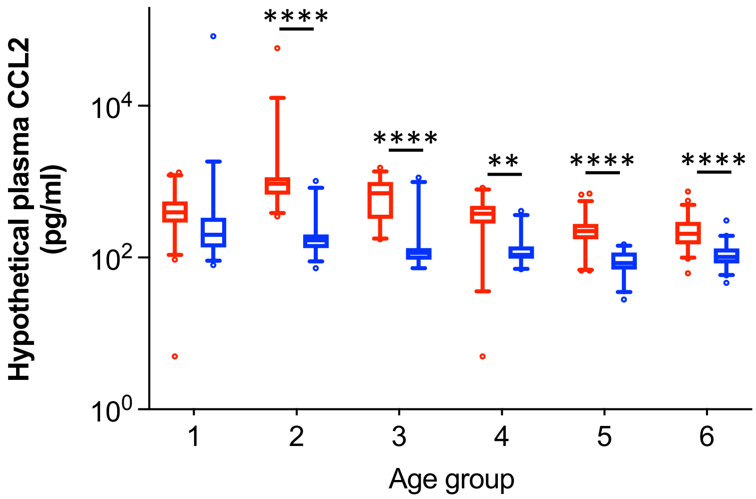

Fig. S3. Comparison of the CCL2 level between normal and affected dogs based on hypothetical plasma values. The measured serum CCL2 concentration was converted to the hypothetical plasma CCL2 concentration using the following formulas: Hypothetical plasma concentration in normal dogs = (Measured serum concentration in normal dogs)/(mean Serum/Plasma ratio in normal dogs) = (Measured serum concentration in normal dogs)/1.62. Hypothetical plasma concentration in affected dogs = (Measured serum concentration in affected dogs)/(mean Serum/Plasma ratio in affected dogs) = (Measured serum concentration in affected dogs)/2.34. Hypothetical plasma levels were log-transformed and analyzed using the linear mixed-effects model. The box spans from the 25th (Q1) to 75th (Q3) percentiles, representing the middle 50% of the data. The line inside the box marks the median (50th percentile). The upper whisker marks the 95th percentile. The lower whisker marks the 5th percentile. Values beyond the 5th and 95th percentiles are considered outliers and depicted as individual circles. Asterisks mark the significance levels. **, $P < 0.01$; ****, $P < 0.0001$.

Disease Models & Mechanisms • Supplementary information

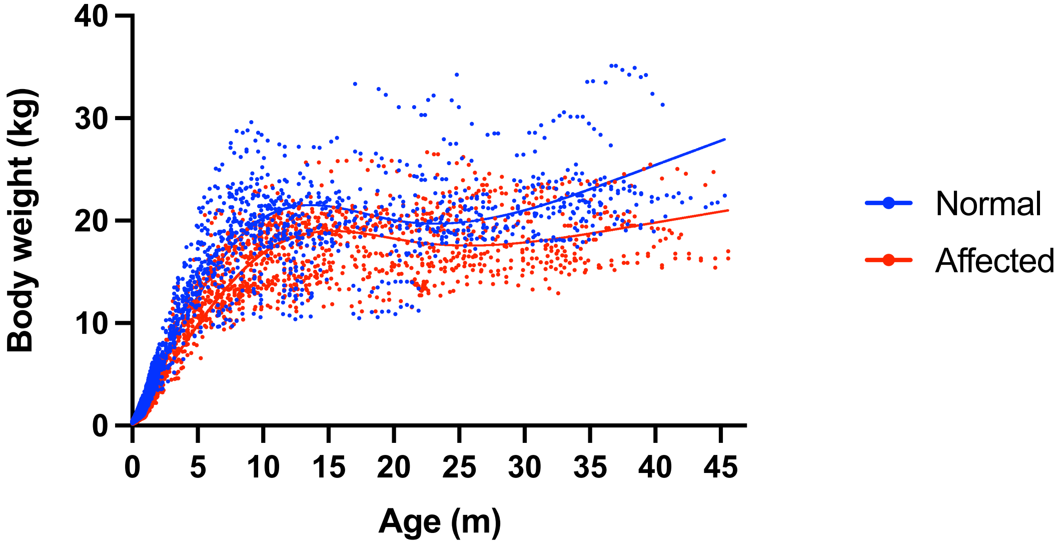

Fig. S4. Body weight of the dogs used in the study. The fitting lines for normal and affected dogs are generated using the Fit Spline tool (smoothing spline, four knots) in the Prism software.

Table S1. Sample with 0 pg/ml and the lowest measurable values

	Serum with 0 pg/ml (%)			Lowest measurable value (pg/ml)	
	Normal	Affected	P value[a]	Normal	Affected
IL-2	62.96	34.31	****	2.27	0.87
IL-6	30.16	16.74	**	0.98	1.89
IL-7	26.98	13.81	**	3.09	2.65
IL-8	0.53	0.00	N/A	69.23	153.58
IL-10	23.81	12.55	**	1.51	0.90
IL-15	50.79	30.13	***	6.07	5.62
IL-18	13.23	7.11	*	1.77	2.31
CCL2	0.00	0.00	N/A	45.61	11.70
CXCL1	0.00	0.84	N/A	23.84	64.26
CXCL10	14.29	13.81	NS	0.84	0.39
GM-CSF	30.69	16.32	**	1.33	1.94
IFN-g	17.46	19.67	NS	0.25	0.32
TNF-a	90.48	84.52	NS	1.64	2.96

a, N/A, not analyzed. NS, not significant. *, P<0.05; **, P<0.01; ****, P<0.0001.

Disease Models & Mechanisms • Supplementary information

Table S2. Descriptive statistics of all 13 cytokines/chemokines (unit: pg/ml)[a]

	Cytokin	N	Min	Max	Mean	SEM	Median	Q1	Q3	95% CI	
										Lower	Upper
Normal	IL-2	189	0.00	1491.05	21.57	8.27	0.00	0.00	9.71	9.96	40.31
	IL-6	189	0.00	14781.39	95.59	78.26	5.63	0.00	12.42	11.09	259.78
	IL-7	189	0.00	2306.45	34.32	12.64	9.76	0.00	24.44	16.99	63.66
	IL-8	189	0.00	97723.74	8886.86	713.36	6020.11	3358.97	11718.33	7635.70	10401.76
	IL-10	189	0.00	11564.83	95.19	61.75	13.71	1.73	27.79	21.83	228.27
	IL-15	189	0.00	3395.80	54.56	19.56	0.00	0.00	31.48	25.45	99.68
	IL-18	189	0.00	1227.11	36.77	8.32	12.50	5.39	28.60	23.07	55.18
	CCL2	189	45.61	134222.36	1030.18	709.27	195.39	154.39	291.40	273.37	2500.16
	CXCL1	189	23.84	5419.12	598.48	42.18	447.31	262.39	771.67	524.59	684.36
	CXCL10	189	0.00	70.58	12.14	1.01	7.15	2.65	17.26	10.15	14.25
	GM-CSF	189	0.00	1145.45	26.35	7.13	5.96	0.00	20.22	14.83	42.91
	IFN-g	189	0.00	484.81	10.63	3.10	1.80	0.92	5.17	5.57	17.48
	TNF-a	189	0.00	685.00	6.60	3.82	0.00	0.00	0.00	1.33	15.92
Affected	IL-2	239	0.00	565.28	28.75	4.84	9.24	0.00	23.03	20.16	38.64
	IL-6	239	0.00	13610.23	106.68	58.34	9.54	3.94	19.05	29.76	234.38
	IL-7	239	0.00	1269.09	50.15	8.94	18.01	8.46	37.86	34.41	68.85
	IL-8	239	153.58	69933.55	16151.25	761.71	12547.78	7838.05	20351.04	14714.10	17676.20
	IL-10	239	0.00	11782.34	74.79	49.22	20.65	10.87	33.11	22.98	175.02
	IL-15	239	0.00	2337.35	88.72	17.67	26.42	0.00	56.90	57.78	124.70
	IL-18	239	0.00	1450.55	61.24	11.41	17.28	9.26	33.08	40.81	85.73
	CCL2	239	11.70	134343.74	1625.80	559.97	814.51	480.46	1517.18	994.57	2811.48
	CXCL1	239	0.00	6409.47	1001.79	50.98	810.96	507.43	1207.96	905.96	1106.16
	CXCL10	239	0.00	104.90	12.42	1.17	6.16	3.82	12.77	10.29	14.77
	GM-CSF	239	0.00	1126.04	43.78	8.34	12.44	5.99	31.17	29.23	60.49
	IFN-g	239	0.00	579.31	9.70	2.82	2.94	0.93	6.66	5.35	16.17
	TNF-a	239	0.00	280.09	9.14	2.40	0.00	0.00	0.00	4.82	14.13

a, Some dogs were sampled multiple times at different ages. These measurements cannot be considered independent. Hence, the numbers shown in this Table cannot be used as population reference values.

Table S3. Comparison of CCL2 results (unit: pg/ml)

	Sample source	N	Measured value (mean)		Measured value (median)	
CCL2 in affected dogs	Serum	17	950.63		587.29	
	Plasma	17	406.85		277.18	
	**Serum/Plasma**		**2.34**		**2.12**	
CCL2 in normal dogs	Serum	21	212.05		201.68	
	Plasma	21	130.71		119.96	
	**Serum/Plasma**		**1.62**		**1.68**	
	**Sample source**	**N**	**Measured serum value (mean)**	**Hypothetical plasma value (mean)***	**Measured serum value (median)**	**Hypothetical plasma value (median)****
CCL2 in age group 1	Affected	42	1050.92	449.77	893.38	421.64
	Normal	33	4776.65	2944.35	310.92	184.94
(0 to < 3m)	**Affected/Normal**		**0.22**	**0.15**	**2.87**	**2.28**
CCL2 in age group 2	Affected	29	6800.07	2906.01	2200.59	1038.02
	Normal	25	345.50	213.27	261.15	155.44
(3 to < 6m)	**Affected/Normal**		**19.68**	**13.63**	**8.43**	**6.68**
CCL2 in age group 3	Affected	20	1643.35	702.29	1729.90	815.99
	Normal	19	279.15	172.31	189.22	112.63
(6 to < 9m)	**Affected/Normal**		**5.89**	**4.08**	**9.14**	**7.24**
CCL2 in age group 4	Affected	18	933.78	399.05	897.61	423.40
	Normal	15	207.65	128.18	173.48	103.26
(9 to < 12m)	**Affected/Normal**		**4.50**	**3.11**	**5.17**	**4.10**
CCL2 in age group 5	Affected	22	518.67	221.65	509.28	240.22
	Normal	22	151.44	93.48	142.55	84.85
(12 to < 24m)	**Affected/Normal**		**3.42**	**2.37**	**3.57**	**2.83**
CCL2 in age group 6	Affected	22	564.12	241.08	465.95	219.79
	Normal	15	165.10	101.91	154.40	91.90
(24 to < 46m)	**Affected/Normal**		**3.42**	**2.37**	**3.02**	**2.39**

*, Hypothetical plasma mean in affected dogs = (Measured serum mean in affected dogs)/(mean Serum/Plasm ratio in affected dogs) = (Measured serum mean in affected dogs)/2.34. Hypothetical plasma mean in normal dogs = (Measured serum mean in normal dogs)/(mean Serum/Plasm ratio in normal dogs) = (Measured serum mean in normal dogs)/1.62.

**, Hypothetical plasma median in affected dogs = (Measured serum median in affected dogs)/(median Serum/Plasm ratio in affected dogs) = (Measured serum median in affected dogs)/2.12. Hypothetical plasma median in normal dogs = (Measured serum median in normal dogs)/(Serum/Plasm ratio in normal dogs) = (Measured serum median in normal dogs)/1.68.

Table S4. Effect of sex on cytokine/chemokine levels

Cytokines/ chemokines	Sex effect		
	Estimate[a]	SEM[b]	P value[c]
IL-2	-0.584	0.477	NS
IL-6	-0.214	0.404	NS
IL-7	-0.295	0.397	NS
IL-8	-0.341	0.240	NS
IL-10	-0.338	0.399	NS
IL-15	-0.840	0.526	NS
IL-18	-0.347	0.323	NS
CCL2	-0.436	0.146	**
CXCL1	-0.114	0.153	NS
CXCL10	-0.566	0.315	NS
GM-CSF	-0.507	0.408	NS
IFN-g	-0.241	0.299	NS
TNF-a	0.114	0.323	NS
CCL2 age group analysis			
< 3m	-0.131	0.281	NS
3m to < 6m	-0.109	0.206	NS
6m to < 9m	-0.515	0.206	*
9m to <12 m	-0.484	0.428	NS
12m to < 24m	0.068	0.133	NS
24m to < 40m	0.020	0.194	NS

a, Estimate is the coefficient in the linear mixed-effects model. Negative values suggest male is lower than female. Positive values suggest male is higher than female.
b, Standard error of mean.
c, NS, not statistically significant. **, P<0.01

Table S5. Effect of age on cytokine/chemokine levels

Cytokines/ chemokines	Age effect		
	Estimate[a]	SEM[b]	P value[c]
IL-2	-0.047	0.016	**
IL-6	-0.042	0.014	**
IL-7	-0.064	0.014	****
IL-8	-0.024	0.005	****
IL-10	-0.092	0.012	****
IL-15	-0.093	0.018	****
IL-18	-0.032	0.012	**
CCL2	-0.033	0.005	****
CXCL1	-0.027	0.006	****
CXCL10	0.034	0.011	**
GM-CSF	-0.061	0.014	****
IFN-g	-0.071	0.010	****
TNF-a	0.001	0.012	NS

a, Estimate is the coefficient in the linear mixed-effects model. The value refers to the change (in log)/month. Negative values suggest age-associated reduction (as dogs get older, the cytokine/chemokine levels go down). Positive values suggest age-associated increase (as dogs get older, the cytokine/chemokine levels go up).

b, SEM, Standard error of mean.

c, NS, statistically significant. *, P<0.05; **, P<0.01; ***, P<0.001; ****, P<0.0001.

Table S6. Relative contribution of each cytokine/chemokine to different principal components.

	PC1	PC2	PC3	PC4	PC5	PC6	PC7	PC8	PC9	PC10	PC11	PC12	PC13
**IL-2**	0.312	-0.133	-0.208	-0.225	0.268	-0.220	0.281	0.615	-0.209	0.341	-0.032	-0.179	-0.144
**IL-6**	0.337	-0.143	0.103	0.040	0.198	-0.052	0.075	-0.688	-0.265	0.424	-0.079	-0.273	0.052
**IL-7**	0.339	-0.155	-0.270	0.006	-0.245	0.019	0.092	-0.137	-0.299	-0.102	-0.139	0.706	-0.286
**IL-8**	0.195	0.495	-0.230	0.220	-0.092	0.187	-0.349	0.082	0.315	0.551	-0.156	0.101	-0.078
**IL-10**	0.234	0.273	0.413	-0.265	-0.124	0.637	0.234	0.093	-0.181	0.040	0.332	0.021	-0.020
**IL-15**	0.349	-0.119	-0.091	-0.022	-0.154	-0.289	0.117	-0.117	0.517	0.005	0.672	0.035	-0.015
**IL-18**	0.331	-0.213	-0.004	0.124	-0.380	0.151	-0.265	0.074	0.022	-0.292	-0.165	-0.536	-0.431
**CCL2**	0.258	0.417	-0.015	0.036	0.236	-0.054	0.521	-0.125	0.330	-0.377	-0.392	-0.065	-0.065
**CXCL1**	0.197	0.520	-0.138	0.177	0.100	-0.298	-0.267	0.016	-0.501	-0.308	0.317	-0.103	0.111
**CXCL10**	0.102	-0.128	0.555	0.722	0.159	-0.105	0.073	0.220	-0.010	0.063	0.035	0.183	-0.124
**GM-CSF**	0.366	-0.170	-0.018	0.105	-0.271	0.062	-0.001	0.173	0.011	-0.070	-0.215	0.000	0.817
**IFN-g**	0.194	0.106	0.552	-0.492	-0.054	-0.420	-0.361	0.026	0.097	0.006	-0.233	0.169	-0.043
**TNF-a**	0.251	-0.240	-0.091	-0.079	0.685	0.345	-0.409	0.012	0.163	-0.240	0.061	0.138	0.036

Table S7. MRMR score

	Score
CCL2	0.330
IL-8	0.076
CXCL10	0.072
IL-7	0.067
CXCL1	0.063
IL-2	0.037
IFN-g	0.031
IL-10	0.030
IL-6	0.024
IL-15	0.024
GM-CSF	0.022
TNF-a	0.019
IL-18	0.009
